# Enterolith Migration From a Jejunal Diverticulum Resulting in Jejunal Perforation

**DOI:** 10.7759/cureus.70451

**Published:** 2024-09-29

**Authors:** Romane Brawermann, Nicolas Claeys, Olivier Cappeliez, Sanjiva Pather, Tom Saliba

**Affiliations:** 1 Surgery, Hôpital de Braine-L'Alleud, Braine-L'Alleud, BEL; 2 Radiology, Hôpital de Braine-L'Alleud, Braine-L'Alleud, BEL; 3 Radiology, Hôpital de Braine-L'Alleud, Braine-L'Alleud, BEL

**Keywords:** enterolith, ileum diverticula, intestinal occlusion, intestinal perforation, sibo

## Abstract

Proximal jejunal enteroliths, a rare form of small bowel pathology, involve calculi formation within the proximal ileum, leading to complications such as bowel obstruction and perforation. Due to their rarity and nonspecific presentation, enteroliths pose diagnostic and management challenges for clinicians. A 73-year-old male with a history of small intestinal bacterial overgrowth was admitted with acute abdominal pain, small bowel obstruction, and hypovolemic haemorrhagic shock. Despite initial stabilization, worsening symptoms led to a CT scan revealing small bowel perforation and enterolith-induced occlusion. Surgery confirmed purulent peritonitis, necessitating resection of the affected bowel segment. Enteroliths can form in diverticula due to bowel content stagnation, causing symptomatic obstruction or perforation. Management typically involves surgical intervention. The prognosis depends on timely diagnosis and treatment to prevent severe complications. Proximal jejunal enteroliths, though rare, should be considered in patients with small bowel obstruction symptoms, particularly those with a history of diverticulosis. Early recognition and appropriate management are crucial for favourable outcomes.

## Introduction

Proximal jejunal enteroliths represent a rare but clinically significant subset of small bowel pathology characterized by the formation of calculi or concretions within the proximal portion of the ileum [1,2].​ Small bowel diverticuli, in which the enterolithiasis forms, are generally asymptomatic with the lesions going unnoticed during radiological examinations and even surgical explorations that are conducted for other causes ​[1].​ When small bower diverticuli are symptomatic, they may cause bloating, epigastric pain, and nausea, but may still go undetected due to the non-specific nature of the symptoms.​ Enterolithiasis in this location can lead to various complications, including small bowel obstruction and perforation.​ As small bowel enteroliths are relatively uncommon compared to those found in the gallbladder or urinary system, their diagnosis and management pose unique challenges to clinicians due to their nonspecific presentation and the pathology being poorly known [1]​.

We present a case of a patient with proximal jejunal enterolithiasis. We explore the challenges encountered in diagnosing and managing this rare condition. With this classic example of enterolithiasis, this report adds to the growing body of knowledge surrounding proximal jejunal enteroliths and enhances awareness among healthcare providers regarding this uncommon but clinically significant entity. 

## Case presentation

A 73-year-old Caucasian male presented to the emergency room (ER) with acute abdominal pain. When he arrived at the hospital, the patient had mottled skin and was hypotensive and tachycardic. The patient had a history of recurrent abdominal pain and small intestinal bacterial overgrowth (SIBO), for which he underwent successive CT scans over the last seven years, alongside multiple gastroenterologist consultations.

We immediately did an arterial blood test showing 1.7 mm/L lactate and his pH was 7.52. The initial blood test showed a normal haemoglobin level but this rapidly decreased in the following hours, dropping from 14.1 g/dl to 10.8 g/dl in under 24 hours.

He was subsequently admitted to the intensive care unit for hemorrhagic hypovolemic shock subsequent to an upper GI tract bleed, in the context of an anticoagulative treatment for paroxysmal atrial fibrillation. The gastroenterologist performed a gastroscopy, discovering fundus gastropathy due to a large hiatal hernia and three superficial ulcers, which he treated with two hemostatic clips. 

Ten days later, despite initial stabilization, the patient's symptoms worsened. He experienced increased abdominal pain, nausea, vomiting, and diarrhoea. Examination revealed abdominal distension and tenderness, with reduced bowel movement sounds. However, his blood tests showed no signs of infection. The patient remained stable and apyretic. The main symptom was unbearable abdominal pain. An abdominal CT scan was performed, revealing a perforation in the small intestine, signs of peritonitis and a large enterolith causing small bowel occlusion.

**Figure 1:**
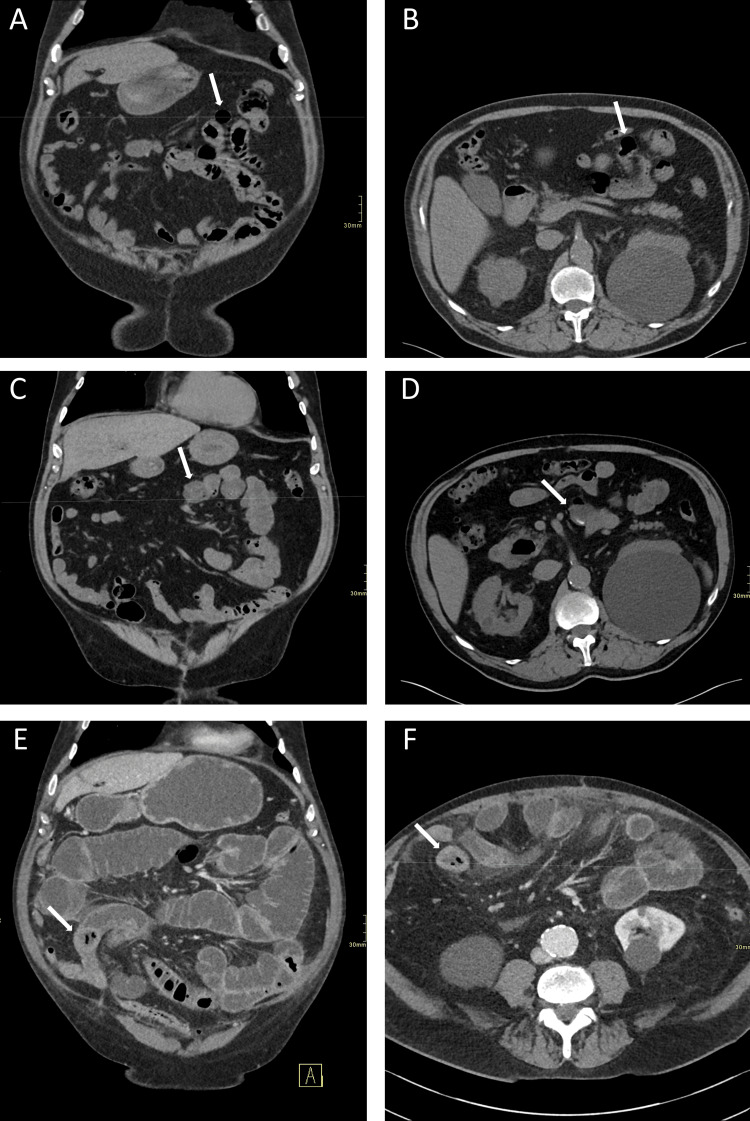
Evolution of the enterolith over seven years Non-contrast enhanced coronal (A) and axial (B) CT scan performed seven years prior to the current presentation showing an empty jejunal diverticulum (arrows). Non-contrast enhanced coronal (C) and axial (D) CT scan performed five years prior to the current presentation showing a jejunal diverticulum containing a partially hyperdense enterolith (arrows). Contrast-enhanced coronal (E) and axial (F) CT scan performed at the current presentation at the emergency room showing the previously seen enterolith, with a slightly hyperdense exterior and air within it, which has migrated and become impacted in the ilium (arrows), causing a proximal occlusion.

This CT scan was compared to the previous CT scans, acquired seven and five years prior to the current presentation, which showed the evolution of the enterolith (Figure 1). These revealed multiple jejunal diverticula in the first CT scan, which were subsequently filled by dense enteroliths in the subsequent scans. When comparing the earlier scans to the one acquired the day of presentation to the ER, one of these enteroliths was found to have been dislodged from a diverticulum and migrated distally before becoming impacted in the small bowel and causing an occlusion and subsequent perforation. 

**Figure 2:**
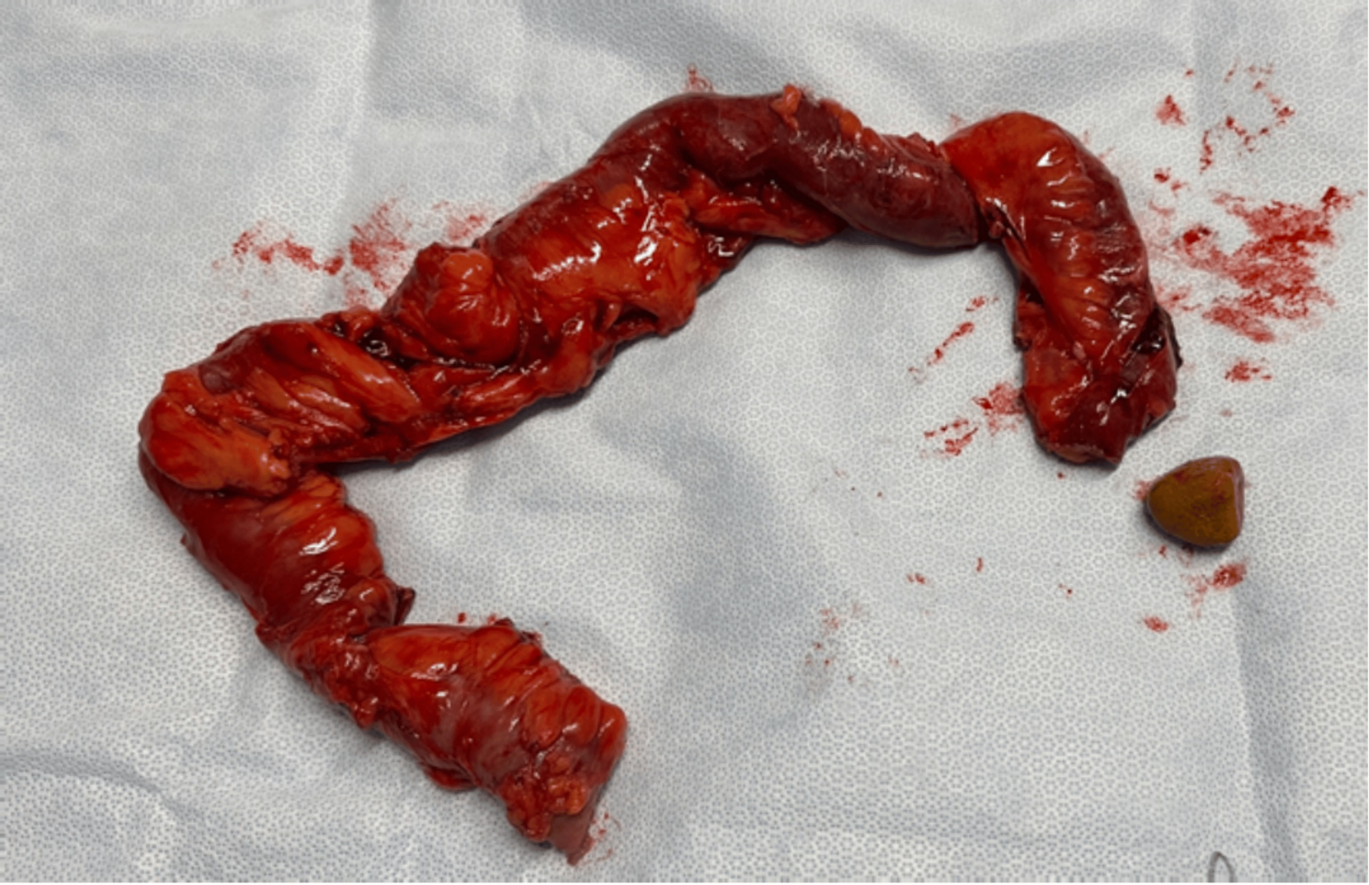
Resected small bowel and enterolith

A nasogastric tube with light drainage aspiration was placed overnight and the patient was prepared for an operation the next morning. During surgery, we discovered purulent peritonitis with a mechanical occlusion of the small intestine and perforation with a large impacted enterolith. Although the surgery was initially planned as a laparoscopic procedure, a laparotomy was necessary. During surgery, the perforated part of the small intestine segment was resected and the enterolith was found (Figure 2).

The abdominal cavity was thoroughly cleaned and drained. The patient’s recovery from the operation was uneventful.

## Discussion

Small bowel diverticula can be caused by small bowel motility disorders, connective tissue disorders, or neuromuscular conduction anomalies ​[3].​ Although mostly asymptomatic, generally incidentally found during radiological exams or laparotomies, patients may present with epigastric pain, abdominal discomfort, or postprandial flatulence ​[1,3,4]. Our patient was typical in this respect as he had experienced non-specific abdominal symptoms for many years, without any specific diagnosis being made. Over time, enteroliths have been known to form in these diverticula due to stagnation of the bowel contents, as was likely the case in our patient ​[3].

The management of proximal jejunal enteroliths depends on various factors, including the patient's clinical presentation, the size and location of the enterolith, and the presence of complications ​[5].​ In cases of symptomatic enteroliths causing obstruction or perforation, surgical intervention is often necessary ​[5]. Surgical options may include enterolithotomy or segmental bowel resection, depending on the extent of bowel involvement and the patient's overall condition [5].​ In asymptomatic patients with incidentally discovered enteroliths, conservative management and close observation may be appropriate [5,6].

The prognosis for patients with proximal jejunal enteroliths largely depends on the timeliness of diagnosis and intervention. Delayed diagnosis or untreated enteroliths can lead to complications such as bowel obstruction, perforation, peritonitis, and sepsis, which significantly negatively impact patient outcomes [7]. However, with prompt recognition and appropriate management, many patients can achieve favourable outcomes with the resolution of symptoms and the prevention of further complications [2,3].

## Conclusions

Proximal jejunal enteroliths represent a rare but clinically significant entity that requires a high index of suspicion for diagnosis. Clinicians should consider enterolithiasis in the differential diagnosis of patients presenting with symptoms of small bowel obstruction, especially without clear signs of more common etiologies and in patients with a history of small bowel diverticulosis. This presentation could also be misdiagnosed as a gallstone ileus. Through a comprehensive diagnostic evaluation and tailored management approach, clinicians can effectively identify and address proximal jejunal enteroliths, ultimately improving patient outcomes and reducing the risk of complications. 
